# Associations of systemic inflammation markers with identification of pulmonary nodule and incident lung cancer in Chinese population

**DOI:** 10.1002/cam4.4606

**Published:** 2022-04-05

**Authors:** Ting Tian, Jing Lu, Wei Zhao, Zhongming Wang, Hai Xu, Yuqing Ding, Wen Guo, Pei Qin, Wenfang Zhu, Ci Song, Hongxia Ma, Qun Zhang, Hongbing Shen

**Affiliations:** ^1^ Department of Epidemiology, Center for Global Health, School of Public Health Nanjing Medical University Nanjing China; ^2^ Jiangsu Key Lab of Cancer Biomarkers, Prevention and Treatment, Collaborative Innovation Center for Cancer Personalized Medicine Nanjing Medical University Nanjing China; ^3^ Health Promotion Center, Jiangsu Province Hospital and the First Affiliated Hospital of Nanjing Medical University Nanjing China; ^4^ Information Department, Jiangsu Province Hospital and the First Affiliated Hospital of Nanjing Medical University Nanjing China; ^5^ Department of Radiology, Jiangsu Province Hospital and the First Affiliated Hospital of Nanjing Medical University Nanjing China; ^6^ Research Units of Cohort Study on Cardiovascular Diseases and Cancers Chinese Academy of Medical Sciences Beijing China

**Keywords:** CT scan, lung cancer, neutrophil‐to‐lymphocyte ratio, platelet‐to‐lymphocyte ratio, pulmonary nodules, systemic immune‐inflammation index

## Abstract

**Objectives:**

Neutrophil‐to‐lymphocyte ratio (NLR), platelet‐to‐lymphocyte ratio (PLR), and systemic immune‐inflammation index (SII), easily accessible systemic inflammation response parameters, were reported to associate with poor lung cancer prognosis. However, research on the effects of these markers on the risk of positive nodules (PNs) and lung cancer is limited.

**Methods:**

Participants in this retrospective study were those who had their first computed tomography (CT) screening at Jiangsu Province Hospital's Health Promotion Center between January 1, 2017 and December 31, 2020. We identified PNs (≥6 mm in diameter) from free text of CT reports and lung cancer from medical records. Multivariate logistic analysis was used to assess the association between NLR, PLR, or SII and PNs or lung cancer.

**Results:**

The detected rate of PNs was 9.60% among the 96,476 participants. Age, smoking and body mass index were possible influencing factors for PNs. We observed linear dose‐effect relationship between NLR, PLR, or SII and PNs (*p*_non‐linear_ > 0.05). Compared with low quintile, participants with top quintiles of NLR, PLR or SII had an increased risk of PNs, with the adjusted ORs of 1.19 (1.11–1.28), 1.11 (1.04–1.19) or 1.11 (1.03–1.18), respectively. Meanwhile, NLR showed the U‐shaped relationship with lung cancer, with adjusted ORs of 1.40 (1.08–1.81) comparing highest NLR quintile to the third quintile. The high PLR and SII showed significantly associated with lung cancer with adjusted ORs of 1.29 (0.99–1.68) and 1.35 (1.04–1.74) comparing to the lowest quintile.

**Conclusions:**

The high levels of systemic inflammation markers were associated with the risk of positive pulmonary nodules and lung cancer, which suggested systemic immune response may be an important pre‐clinical feature for the early identification of diseases.

## Graphical abstract

In this large sample of Chinese populations, the identification rate of positive pulmonary nodules (≥6 mm) was 9.60%. We also found that elevated Neutrophil‐to‐lymphocyte ratio (NLR), platelet‐to‐lymphocyte ratio (PLR) and systemic immune‐inflammation index (SII) were associated with increased risk of positive nodules identification. Then, high levels of these three markers showed significantly associated with lung cancer.

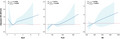

## 1 INTRODUCTION

Lung cancer is currently one of the most frequently diagnosed cancer worldwide, as well as the leading cause of cancer‐related mortality.^1^ Implementing early detection‐screening is a critical step toward reducing lung cancer‐related deaths and improving survival.^2^ Efforts toward this purpose have been ongoing for a long time. Historically, previous studies showed that computes tomography (CT) is more sensitive than chest radiography (CR) in identifying the pulmonary nodules and lung cancer.^3^, ^4^, ^5^

Increasingly widespread use of CT in clinical practice has resulted in a sharp rise in incidental findings of pulmonary nodules.^6^ In 2003, one Mayo Clinic study with low‐dose CT found pulmonary nodules in 69% of participants.^7^ Approximately 30% of CT scans with one or more nodules were found in a study conducted in the United States with over 200,000 adult participants.^8^ However, a high rate of benign nodules were detected, which raises concerns of overdiagnosis. Nodules <6 mm in diameter do not require regular follow‐up due to the low risk associated with such nodules.^9^ The International Early Lung Cancer Action Program (I‐ELCAP) reported that lung cancer diagnosis rates were 0.3% and 10.06% for nodule sizes of <6 and ≥6 mm, respectively.^10^ The definition of a positive result has changed from the threshold of ≤5 mm by 2020^11^ to <6 mm by 2021^12^ in the National Comprehensive Cancer Network (NCCN) Guidelines. Thus, these highlight a need to assess the factor on occurrence of positive pulmonary nodules.

Systemic inflammation is well acknowledged to have a key role in carcinogenesis, progression and prognosis of cancer.^13^, ^14^, ^15^ As a result, several inflammatory markers have been identified. Detailed research has been done on the neutrophil to lymphocyte ratio (NLR),^16^ the platelet‐to‐lymphocyte ratio (PLR)^17^ and systemic immune‐inflammation index (SII),^18^ all of which reflect individual’s overall inflammatory status. Previous research in lung cancer has consistently focused on the prognostic value of these parameters in patients who have already been diagnosed.^16^, ^17^, ^19^, ^20^, ^21^, ^22^ The evidence showed that the elevated three values have been associated with a poor prognosis. Recently, a cohort study found that NLR has been linked to an increased risk of lung cancer mortality.^23^ Few studies, however, have examined the relationship between these values and pulmonary nodules or lung cancer risk in healthy subjects.

Therefore, we conducted a retrospective study of asymptomatic participants who voluntarily participated in health examination and underwent CT in China. Firstly, we evaluated the initial detection rate of positive nodules in populations with different characteristics. Then, we evaluated whether NLR, PLR or SII was associated with identification of positive nodules. Furthermore, we also examined whether or not there were links between these markers and lung cancer risk.

## 2 MATERIAL AND METHODS

### 2.1 Data source

In China, employees are required to undergo annual or biennial health examination. Health Promotion Center of Jiangsu Province Hospital mainly provide comprehensive care for examinees in Nanjing, where about 75% were employees of various companies or local governmental agencies. This center implemented an electronic medical record system in 2014, which stores data on sociodemographic, diagnoses, laboratory tests, comorbidities, medical history, and radiology and so on. Structured data and free‐text radiology reports were retrieved from this electronic medical system and used in this study.

The approval was obtained from ethics committees at Jiangsu Province Hospital and the First Affiliated Hospital of Nanjing Medical University. Analyses were conducted using anonymized and de‐identified data, and thus informed consent was not required.

### 2.2 Study design and participants

This was a retrospective study of adults over the age of 18 who voluntarily underwent a comprehensive health examination and chest CT imaging at Health Promotion Center of Jiangsu Province Hospital between Jan 1, 2017 and Dec 31, 2020 (*N* = 112,629). In this retrospective analysis, we extracted data from the participants’ first chest CT as baseline data, even if the participants underwent more than once chest CT during this period. Subsequently, we used a structured processing method^24^ to scan the free text of radiology reports on their chest CT scans to identify reports that mentioned the presence of one or more pulmonary nodules measuring ≥6 mm in diameter. Given that the complex semantic structures may be imperfect matched, we also review manually to determine the positive nodules.

We excluded 16,153 participants who met at least one of the following exclusion criteria mentioned below: (1) a history of lung cancer or lung surgery (*N* = 440); (2) a history of other malignancy (*N* = 4175); (3) missing data on smoking status, body mass index (BMI), platelets, neutrophil, lymphocyte, and monocyte (*N* = 11,495); (4) lung mass (>3 cm in diameter) (*N* = 43). In total, there were 96,476 participants who were eligible to take part in this study (Figure S1).

### 2.3 Data collection

Demographic characteristics, such as age, gender, smoking status, and medical history (hypertension and diabetes) were self‐reported by participants. For this study, participants were categorized into never‐smokers and ever‐smokers. Participants were classified as never‐smokers if they had never smoked or if they had smoked <100 cigarettes in their lifetime.^25^ Former smokers and current smokers were classified as ever‐smokers.

For each participant, BMI was calculated as weight (kilogram)/height^2^ (square meter). Blood pressure (BP) was recorded by trained nurses with an electronic sphygmomanometer after at least 5 min of seated rest. Hypertension was defined as a self‐reported history of hypertension, or BP ≥140/90 mm Hg.^26^ Diabetes were defined as a self‐reported history of diabetes, and/or glycated hemoglobin (HbA_1_c) ≥6.5%, or fasting blood glucose (FBG) ≥7.0 mmol/L.^27^ Diagnosed lung disease included asthma, chronic obstructive pulmonary diseases, chronic bronchitis, bronchiectasis, bullae, emphysema and so on.

Blood samples were taken from participants after at least a 10‐h fast. White blood cell count, neutrophils, lymphocytes, platelet, eosinophil, and basophil count were measured using the same automatic hematology analyzer. NLR and PLR were calculated by dividing the absolute neutrophil (N, ×10^9^/L) and platelet count (P, ×10^9^/L) by the lymphocyte count (L, ×10^9^/L), respectively. The SII was defined as follows: SII = P×N/L.^28^

### 2.4 Outcomes

The primary outcomes were occurrence of positive pulmonary nodules risk and lung cancer risk. According to the NCCN guideline,^12^ a positive nodule detected by CT was defined as any noncalcified nodule with diameter of ≥6 mm in this study. The cancer status information was acquired from complete inpatient and outpatient medical records. Lung cancer cases were confirmed by the 10th Revision of International Classification of Diseases (ICD‐10) code C34. Information on lung cancer was available up till December 1, 2021.

### 2.5 Statistical analysis

Demographic characteristics of the study participants were calculated and compared among groups. The characteristics of participants were summarized using descriptive statistics by NLR, PLR and SII quintiles. NLR was categorized into quintiles as follows: Q1 (<1.28), Q2 (1.28‐ < 1.57), Q3 (1.57‐ < 1.88), Q4 (1.88‐ < 2.33) and Q5 (≥2.33); PLR was categorized into quintiles as follows: Q1 (<87.11), Q2 (87.11– < 104.92), Q3 (104.92– < 122.77), Q4 (122.77– < 147.40) and Q5 (≥147.40). SII was categorized into quintiles as follows: Q1(<262.06), Q2 (262.06– < 336.92), Q3 (336.92– < 418.21), Q4 (418.21– < 540.65), Q5 (≥540.65). As appropriate, data are presented as mean (standard deviation, SD) or as number (percentage). T‐tests for continuous variables and Chi‐squared tests for categorical variables were used to analyze baseline characteristics.

The endpoints were the occurrence of positive pulmonary nodules and incident lung cancer. Data of their first screen (baseline data) were used to evaluate the associations between three markers and positive nodule or lung cancer. We computed the odds ratios (ORs) and 95% confidence intervals (CIs) for positive nodule or incident lung cancer using logistic regression models to compare each category of markers among overall populations. Further adjustments were made to the multivariable model for age (continuous variables), gender (female, male), smoking status (never, ever), BMI (continuous variables), diagnosed hypertension (no, yes), diagnosed diabetes (no, yes), and diagnosed lung diseases (no, yes). To assess the concentration‐response relationship between markers and positive nodules identification, we modeled NLR, PLR, or SII as restricted cubic splines with knots at the quartiles of the sample distribution.

To investigate the potential source of heterogeneity, subgroup analyses were performed by age, gender, smoking status, BMI. Using likelihood ratio tests, we compared models with and without multiplicative interaction terms to see if there were any interactions between NLR, PLR, or SII categories and subgroup characteristics.

All *p*‐values were two‐sided and *p* < 0.05 was considered statistically significant. Analyses in this study were performed using STATA software version 16 (STATA Corp) and R software version 3.6.1 (R Foundation for Statistical Computing, http://www.R‐project.org/).

## 3 RESULTS

### 3.1 Study participants

A total of 96,476 participants underwent their initial chest CT screening included in this study, including 9264 participants (9.60%) with incidental positive pulmonary nodules. The baseline characteristic of participants was listed in Table 1. At baseline, the mean (SD) age of all the participants was 46.71 (13.83) years, and the mean age of participants with positive nodules was significantly greater than those without positive nodules (51.12 vs. 46.24 years, *p* < 0.001). Male participants account for about 60.62% of the total populations, and the gender distribution was similar between groups. The proportion of ever‐smokers with positive nodules was larger than the other group (24.85% vs. 23.11%, *p* < 0.001). Compared with participants without positive nodules, those with positive nodules more likely to have underlying diseases (i.e., hypertension [36.36%], diabetes [10.22%], lung diseases [0.86%]). The mean values of platelets, neutrophil, lymphocyte, monocyte counts were differed significantly between groups. The mean values of NLR, PLR and SII in participants with positive nodules were higher than the other group (NLR: 1.93 ± 0.87 vs. 1.86 ± 0.78, *p* < 0.001; PLR: 120.18 ± 42.00 vs. 119.35 ± 40.06, *p* = 0.061; SII: 423.61 ± 224.53 vs. 416.54 ± 209.85). Baseline characteristics are shown by NLR quintiles, PLR quintiles and SII quintiles (Tables S1, S2 and S3).

**TABLE 1:** Baseline characteristics of participants with positive pulmonary nodules detected by CT screening

	Total (*N* = 96,476)	Without positive nodules (*N* = 87,212)	With positive nodules (*N* = 9,264)	*p* value
Age (years)	46.71 ± 13.83	46.24 ± 13.68	51.12 ± 14.44	<0.001
<50	60,203 (62.40)	55,622 (63.78)	4581 (49.45)	<0.001
≥50	36,273 (37.60)	31,590 (36.22)	4683 (50.55)	
Gender				
Female	37,990 (39.03)	34,374 (39.41)	3616 (39.03)	0.475
Male	58,486 (60.62)	52,838 (60.59)	5648 (60.97)	
BMI (kg/m^2^)				
≤18.4	2324 (2.41)	2097 (2.40)	227 (2.45)	<0.001
18.5–22.9	33,233 (34.45)	30,219 (34.65)	3014 (32.53)	
≥23.0	60,919 (63.14)	54,896 (62.95)	6023 (65.02)	
Smoking status				
Never	74,023 (76.73)	67,061 (76.89)	6962 (75.15)	<0.001
Ever	22,453 (23.27)	20,151 (23.11)	2302 (24.85)	
SBP (mm Hg)	126.59 ± 17.81	126.35 ± 17.70	128.83 ± 18.71	<0.001
DBP (mm Hg)	77.44 ± 11.31	77.40 ± 11.32	77.83 ± 11.31	<0.001
Diagnosed hypertension				
No	67,207 (69.66)	61,311 (70.30)	5896 (63.64)	<0.001
Yes	29,269 (30.34)	25,901 (29.70)	3368 (36.36)	
Diagnosed diabetes				
No	89,130 (92.39)	80,813 (92.66)	8317 (89.78)	<0.001
Yes	7346 (7.61)	6399 (7.34)	947 (10.22)	
Diagnosed lung disease				
No	95,951 (99.46)	86,767 (99.49)	9184 (99.14)	<0.001
Yes	525 (0.54)	445 (0.51)	80 (0.86)	
Platelets (×10^9^/L)	223.80 ± 55.05	224.26 ± 54.98	219.48 ± 55.59	<0.001
WBC count (×10^9^/L)	6.07 ± 1.71	6.07 ± 1.70	6.07 ± 1.80	0.804
Neutrophil count (×10^9^/L)	3.50 ± 1.20	3.49 ± 1.20	3.52 ± 1.17	0.025
Lymphocyte count (×10^9^/L)	2.00 ± 0.79	2.00 ± 0.75	1.96 ± 1.07	<0.001
Monocyte count (×10^9^/L)	0.40 ± 0.14	0.40 ± 0.14	0.41 ± 0.13	<0.001
NLR	1.87 ± 0.79	1.86 ± 0.78	1.93 ± 0.87	<0.001
PLR	119.43 ± 40.25	119.35 ± 40.06	120.18 ± 42.00	0.061
SII	417.22 ± 211.32	416.54 ± 209.85	423.61 ± 224.53	0.002

*Note*: Data are number (percentage) or mean ± standard deviation.

Abbreviations: BMI, body mass index; SBP, Systolic blood pressure; DBP, Diastolic blood pressure; WBC, White blood cell; NLR, neutrophil‐to‐lymphocyte ratio; PLR, platelet to lymphocyte ratio; SII, systemic immune‐inflammation index.

### 3.2 Initial rate of positive nodules identification

**FIGURE 1:**
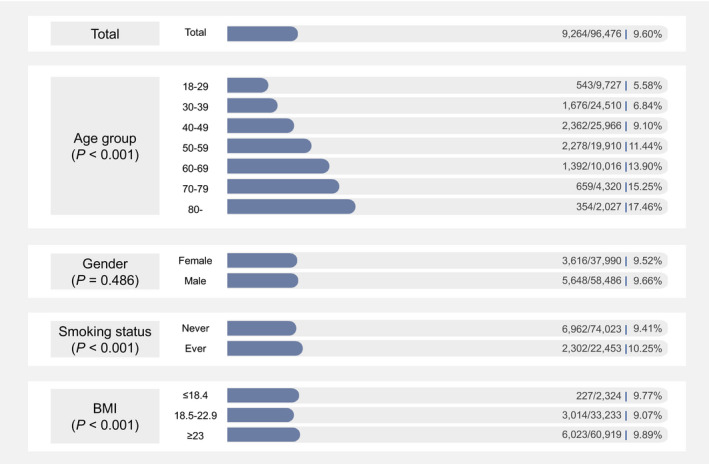
Initial identification rate of positive nodules in populations with different characteristics at baseline

Figure 1 shows the initial identification rate of positive nodules among populations with different characteristics at baseline. Among 96,476 participants, 9.60% were identified with positive nodules. The rates of positive nodules identification appeared to increase over age, from 5.58% for aged 18–29 years to 17.46% for those aged over 80 years (*p* < 0.001). Ever‐smokers have higher detection rates of positive nodule than never‐smokers (10.25% vs. 9.41%, *p* < 0.001). The rates were 9.52% and 9.66% for adult women and men, respectively (*p* = 0.486). Participants with normal BMI (18.5–22.9 kg/m^2^) have slightly low detection rate (9.07%). When a multivariable model was used to further evaluate the associations, age, BMI and smoking were found to be possible influencing factors for positive pulmonary nodules (Figure S2).

### 3.3 Associations of NLR or PLR and positive nodules identification

**FIGURE 2:**
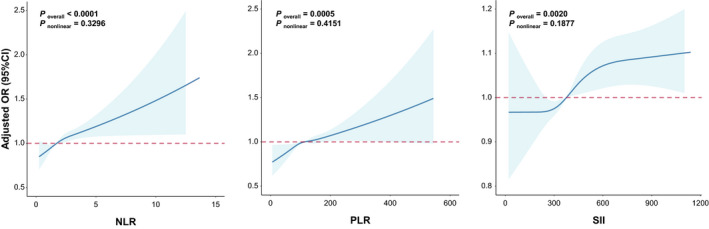
Linear relationships between positive nodules identification and three related inflammation response markers. The curves represent adjusted odds ratios (ORs) based on restricted cubic splines with the knots at the 25th, 50th, 75th, and 95th percentiles of their sample distribution. Logistic regression models were used to estimate OR (95% CI). Multivariable model was adjusted for age, gender, smoking status, BMI, diagnosed hypertension, diagnosed diabetes, and diagnosed lung diseases

As shown in Figure 2, we observed obviously linear dose‐effect relationship between NLR (*p*_non‐linear_ = 0.3296), PLR (*p*_non‐linear_ = 0.4151) or SII (*p*_non‐linear_ = 0.1877) and positive nodules identification with the restricted cubic spline analysis. Then, we grouped the overall participants into five levels according to quintiles. After adjusting for age, gender, BMI, smoking status, diagnosed hypertension, diagnosed diabetes and diagnosed lung diseases, the adjusted OR (95% CI) for positive nodules identification in quintiles 2, 3, 4 and 5 of NLR when compared to the lowest quintile were 1.04 (0.96–1.11), 1.07 (1.00–1.15), 1.08 (1.01–1.16) and 1.19 (1.11–1.28), respectively (*p* for trend <0.001, Table 2). When compare with lowest quintile of PLR, the adjusted OR of quintiles 2, 3, 4 and 5 for positive nodules identification were 1.04 (0.98–1.12), 1.06 (0.99–1.14), 1.13 (1.05–1.21), and 1.11 (1.04–1.19), respectively (*p* for trend <0.001, Table 2). Similarly, the higher SII was associated with the occurrence of positive nodules (OR = 1.09 (1.02–1.17) and 1.11 (1.03–1.18) for quintiles 4 and 5, respectively; *p* for trend <0.001, Table 2).

**TABLE 2:** Odds ratios (95% CIs) for positive nodules by neutrophil‐lymphocyte ratio (NLR) quintile, platelets‐lymphocyte ratio (PLR) quintiles and systemic immune‐inflammation index (SII) among overall populations

	Participants without positive nodules	Participants with positive nodules	Adjusted OR (95% CI)	*p* value
NLR quintiles				
Q1 (<1.28)	17,597	1698	Ref	
Q2 (1.28–1.57)	17,544	1751	1.04 (0.96–1.11)	0.336
Q3 (1.57–1.88)	17,485	1811	1.07 (1.00–1.15)	0.063
Q4 (1.88–2.33)	17,439	1856	1.08 (1.01–1.16)	0.035
Q5 (≥2.33)	17,147	2148	1.19 (1.11–1.28)	<0.001
*p* for trend			<0.001	
PLR quintiles				
Q1 (<87.11)	17,426	1869	Ref	
Q2 (87.11–104.92)	17,476	1819	1.04 (0.98–1.12)	0.215
Q3 (104.92–122.77)	17,497	1799	1.06 (0.99–1.14)	0.098
Q4 (122.77–147.40)	17,405	1890	1.13 (1.05–1.21)	0.001
Q5 (≥147.40)	17,408	1887	1.11 (1.04–1.19)	0.002
*p* for trend			<0.001	
SII quintiles				
Q1 (<262.06)	17,446	1849	Ref	
Q2 (262.06–336.92)	17,534	1761	0.99 (0.92–1.06)	0.741
Q3 (336.92–418.21)	17,477	1819	1.04 (0.97–1.12)	0.222
Q4 (418.21–540.65)	17,403	1892	1.09 (1.02–1.17)	0.013
Q5 (≥540.65)	17,352	1943	1.11 (1.03–1.18)	0.003
*p* for trend			<0.001	

Logistic regression models were used to estimate odds ratios (ORs) and 95% confidence intervals (CIs). Multivariable model was adjusted for age, gender, smoking status, BMI, diagnosed hypertension, diagnosed diabetes and diagnosed lung diseases.

Subgroup analyses according to population characteristics showed that the association between NLR or SII and positive nodules significantly differed by age group (*p* for interaction = 0.0062 and 0.0112, respectively; Tables S4 and S6). In aged <50 years, the top NLR and SII quintile groups showed the OR (95% CI) were 1.16 (1.06–1.28) and 1.05 (0.95–1.16), whereas in aged ≥50 years, the OR (95% CI) of the top quintile groups were 1.30 (1.18–1.43) and 1.17 (1.06–1.28). The associations between PLR and positive nodules were more significantly among aged >50 years, males, never‐smokers and overweight populations (Table S5). No significant differences were observed between subgroups for PLR (*p* for interaction >0.05, Table S5).

### 3.4 Associations of NLR or PLR and incident lung cancer

**FIGURE 3:**
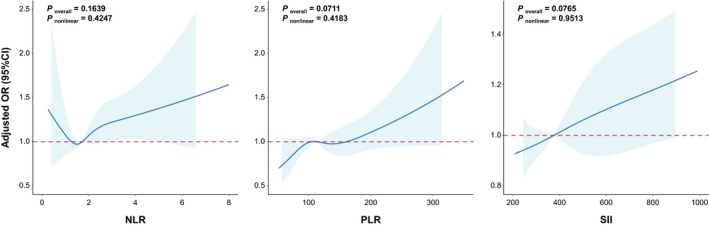
Linear relationships between incident lung cancer and three related inflammation response markers. The curves represent adjusted odds ratios (ORs) based on restricted cubic splines with the knots at the 25th, 50th, 75th, and 95th percentiles of their sample distribution. Logistic regression models were used to estimate OR (95% CI). Multivariable model was adjusted for age, gender, smoking status, BMI, diagnosed hypertension, diagnosed diabetes, and diagnosed lung diseases

Up to date of December 1, 2021, 569 of all participants were diagnosed as lung cancer cases. Table S7 showed the distribution of the baseline characteristics of lung cancer cases and controls. Then, we examined the relationship between baseline NLR, PLR or SII and lung cancer risk. As shown in Figure 3, NLR showed a U‐shaped association with lung cancer, but PLR and SII showed the linear dose‐effect relationship with lung cancer (*p*_overall_ = 0.0711 and 0.0765, respectively). As shown in Table 3, the adjusted ORs (95% CI) for lung cancer in NLR quintiles 1, 2, 4 and 5 when compared to the quintile 3 were 1.15 (0.88–1.52), 1.12 (0.85–1.48), 0.98 (0.74–1.30), and 1.40 (1.08–1.81), respectively (*p* for quadratic term = 0.0145). Higher PLR and SII quintiles showed significant association with lung cancer risk when compared to the lowest PLR quintile group (OR = 1.29 (0.99–1.68) and 1.35 (1.04–1.74) for the highest PLR and SII quintile, Table 3). Subgroup analysis revealed no significant difference between subgroups (*p* for interaction >0.05, Tables S8/S9/S10)

**TABLE 3:** Odds ratios (95% CIs) for lung cancer by neutrophil‐lymphocyte ratio (NLR) quintiles, platelets‐lymphocyte ratio (PLR) quintiles and systemic immune‐inflammation index (SII) among overall populations

	Participants without lung cancer (*N*)	Participants with lung cancer (*N*)	Adjusted OR (95% CI)	*p* value
NLR quintiles				
Q1 (<1.28)	19,183	112	1.15 (0.88–1.52)	0.309
Q2 (1.28–1.57)	19,188	107	1.12 (0.85–1.48)	0.410
Q3 (1.57–1.88)	19,200	96	Ref	
Q4 (1.88–2.33)	19,198	97	0.98 (0.74–1.30)	0.867
Q5 (≥2.33)	19,138	157	1.40 (1.08–1.81)	0.010
*p* for quadratic term			0.0145	
PLR quintiles				
Q1 (<87.11)	19,190	105	Ref	
Q2 (87.11–104.92)	19,184	111	1.17 (0.89–1.53)	0.260
Q3 (104.92–122.77)	19,185	111	1.19 (0.91–1.56)	0.205
Q4 (122.77–147.40)	19,184	111	1.18 (0.90–1.54)	0.241
Q5 (≥147.40)	19,164	131	1.29 (0.99–1.68)	0.057
*p* for trend			0.0861	
SII quintiles				
Q1 (<262.06)	19,188	107	Ref	
Q2 (262.06–336.92)	19,189	106	1.08 (0.82–1.41)	0.592
Q3 (336.92–418.21)	19,180	116	1.22 (0.94–1.59)	0.140
Q4 (418.21–540.65)	19,191	104	1.07 (0.82–1.41)	0.609
Q5 (≥540.65)	19,159	136	1.35 (1.04–1.74)	0.023
*p* for trend			0.0455	

Logistic regression models were used to estimate odds ratios (ORs) and 95% confidence intervals (CIs). Multivariable model was adjusted for age, gender, smoking status, BMI, diagnosed hypertension, diagnosed diabetes and diagnosed lung diseases.

## 4 DISCUSSION

In this large retrospective analysis of Chinese population participating in health‐screening exams, elevated NLR, PLR or SII were associated with increased risk of positive nodules identification. Furthermore, U‐shaped association was observed between NLR and lung cancer. However, the high NLR, PLR, and SII showed significantly associated with incident lung cancer. These findings may suggest that systemic inflammation may associated with the occurrence of positive nodules and lung cancer risk.

Recently, with the increased emphasis on routine health examinations and popularity of chest CT scans, numerous subjects with incidental pulmonary nodules has gradually increased.^8^ An incidental pulmonary nodule can create anxiety for patients due to the asymmetry of patient knowledge and the fear of lung cancer. According to the previous study, almost 26.32% of the pulmonary nodules were detected during a routine health examination, the majority of which were benign.^29^ To reduce false‐positive results, the size threshold for a positive screening was increased. The recommended nodule size threshold for a positive screening has been updated to 6 mm in diameter by the NCCN guidelines.^12^ Thus, we should pay more attention on the occurrence of positive nodule. In the National Lung Screening Trial (NLST) research, nodules with >4 mm in diameter were incidentally identified in an estimated 27.3% by CT.^30^ Another study also showed that about 11.18% of the participants who underwent health examination had nodules with ≥5 mm.^29^ In this current study, we also found that the initial detection rate of ≥6 mm nodules was 9.60% among health‐examination populations. We also found that increased age, overweight and smoking were all associated with positive nodules.

NLR, PLR, and SII are markers of systemic inflammation response. There has been no prior research that explored the relationship between these three markers and the identification of positive nodules. Fortunately, this study with large sample size firstly examined the association between these markers and positive nodules identification in overall populations. Our study demonstrated that the high NLR or PLR may associate with high risk of positive nodules. More than 95% of detected nodules are benign and the most common cause is a previous infection.^9^, ^31^ In this case, we also examined the association between these markers and incident lung cancer. We found that the high levels of these markers were significantly associated with lung cancer risk, which demonstrated that the systemic immune response may be an important pre‐clinical feature in the development of lung cancer.

To the best of our knowledge, inflammation microenvironment plays a crucial role in carcinogenesis.^32^ Neutrophils and platelets have been reported to relate with inflammation of tumor microenvironment. Neutrophils are recruited with cytokines and then enhance carcinogenesis and cancer progression.^33^ Platelets released factors that aid tumor growth, invasion and angiogenesis.^34^ Meanwhile, lymphocytes have a vital part in the production of cytokines, which limit cancer cell growth and cause cytotoxic cell death.^35^ Previous meta‐analysis showed that having a high NLR, PLR, or SII was linked to an worse overall survival in many cancers, including lung cancer.^16^, ^22^, ^36^ However, few research has looked into the association between these markers and lung cancer development. Sanchez‐Salcedo et al.^37^ revealed that baseline NLR and PLR were not significant predictors of lung cancer. However, based on a cohort analysis of 527,124 Korean individuals, Kang et al. demonstrated that higher NLR was related with an increased risk of lung cancer death.^23^ Recently, one study based UK biobank observed positive association with risk for lung cancer with NLR, PLR, and SII.^28^ In our study, the U‐shaped association between NLR and lung cancer was observed. Meanwhile, high level of NLR was significantly associated with lung cancer. High PLR and SII showed significant association with lung cancer, which is in agreement with the observations in the Dutch study^38^ and UK Biobank study.^28^

Several limitations should be noted in this current study. Firstly, although we used structured processing method and manual review for nodule identification, some ambiguous or nonspecific terminology in CT reports may have resulted in missed incidental nodules since no images were reviewed. This should be considered when interpreting our estimates. Secondly, smoking status was self‐reported and was therefore subject to reporting biases. Due to the lack of standard questionnaire, participants did not report the detailed information of smoking exposure such as duration of cessation, cigarette consumptions. These problems should be improved in the further study. Thirdly, despite the large‐scale populations, the included participants were restricted in the single center. The lung cancer cases should be followed up for a longer time. Finally, this study was a population‐based cross‐sectional analysis. To better explore the causal effect, further robust epidemiological evidence, like cohort study, is urgently needed.

In conclusion, in this large retrospective study of healthy populations, high NLR, PLR, and SII, easily accessible systemic inflammation response parameters, were associated with positive nodules and incident lung cancer. These results may indicate that systemic immune response may be an important pre‐clinical feature for the early identification of disease.

## CONFLICT OF INTEREST

None.

## AUTHORS' CONTRIBUTIONS

Dr Qun Zhang and Dr Hongbing Shen had full access to all the data in the study and takes responsibility for the integrity of the data and the accuracy of the data analysis. Concept and design: Ting Tian, Jing Lu; Acquisition, analysis, or interpretation of data: Wei Zhao, Zhongming Wang, Hai Xu, Yuqing Ding, Wen Guo; Pei Qin; Wenfang Zhu; Ci Song; Drafting of the manuscript: Ting Tian, Jing Lu; Critical revision of the manuscript for important intellectual content: Hongbing Shen, Qun Zhang, Hongxia Ma, Ci Song; Statistical analysis: Ting Tian; Obtained funding: Hongbing Shen, Qun Zhang, Ci Song, Ting Tian; Administrative, technical, or material support: All authors;

## ETHICAL STATEMENT

The approval of this study was obtained from ethics committees at Jiangsu Province Hospital and the First Affiliated Hospital of Nanjing Medical University.

## INFORMED CONSENT IN STUDIES

Analyses were conducted using anonymized and de‐identified data, and thus informed consent was not required.

## Supporting information


Appendix S1

Figure S1

Figure S2

## Data Availability

The data are available from the corresponding author upon reasonable request.
