# Supplementary material for: Associations of systemic inflammation markers with identification of pulmonary nodule and incident lung cancer in Chinese population
Source: Cancer Med. 2022 Apr 5;11(12):2482–91. doi: 10.1002/cam4.4606 (PMC9189452; doi:10.1002/cam4.4606)
Supplement: Supplementary file 1 — Appendix S1 [file CAM4-11-2482-s001.docx]

**Table S1. Baseline characteristics of study participants by neutrophil-lymphocyte ratio (NLR) quintiles.**

| Characteristics | Overall | NLR quintiles | | | | |
| --- | --- | --- | --- | --- | --- | --- |
|  |  | Q1 (<1.28) | Q2 (1.28-1.57) | Q3 (1.57-1.88) | Q4 (1.88-2.33) | Q5 (≥2.33) |
| Number | 96,476 | 19,295 | 19,295 | 19,296 | 19,295 | 19,295 |
| Age (years) | 46.71 ± 13.83 | 45.79 ± 13.58 | 45.81 ± 13.43 | 46.03 ± 13.43 | 46.79 ± 13.59 | 49.12 ± 14.79 |
| Male (%) | 58,486 (60.62) | 11,824 (61.28) | 11,987 (62.12) | 11,842 (61.37) | 11,574 (59.98) | 11,259 (58.35) |
| BMI (kg/m^2^) | 24.22 ± 3.52 | 24.03 ± 3.28 | 24.26 ± 3.29 | 24.32 ± 4.31 | 24.30 ± 3.28 | 24.17 ± 3.31 |
| Ever-smokers (%) | 22,453 (23.27) | 4,049 (20.98) | 4,442 (23.03) | 4,607 (23.88) | 4,634 (24.02) | 4,721 (24.47) |
| Diagnosed hypertension (%) | 29,269 (30.34) | 5,167 (26.78) | 5,421 (28.10) | 5,764 (29.87) | 6,034 (31.27) | 6,883 (35.67) |
| Diagnosed diabetes (%) | 7,346 (7.61) | 1,124 (5.83) | 1,211 (6.28) | 1,421 (7.36) | 1,532 (7.94) | 2,058 (10.67) |
| Diagnosed lung disease (%) | 525 (0.54) | 95 (0.49) | 105 (0.54) | 100 (0.52) | 87 (0.45) | 138 (0.72) |
| Platelets (×10^9^/L) | 223.80 ± 55.05 | 222.39 ± 52.56 | 224.63 ± 53.22 | 224.25 ± 53.58 | 225.11 ± 55.52 | 222.62 ± 60.00 |
| WBC count (×10^9^/L) | 6.07 ± 1.71 | 5.56 ± 1.73 | 5.80 ± 1.94 | 5.96 ± 1.35 | 6.21 ± 1.41 | 6.81 ± 1.75 |
| Neutrophil count (×10^9^/L) | 3.50 ± 1.20 | 2.56 ± 0.67 | 3.07 ± 1.04 | 3.41 ± 0.79 | 3.80 ± 0.88 | 4.64 ± 1.34 |
| Lymphocyte count (×10^9^/L) | 2.00 ± 0.79 | 2.44 ± 1.25 | 2.15 ± 0.76 | 1.98 ± 0.46 | 1.83 ± 0.42 | 1.58 ± 0.41 |
| Monocyte count (×10^9^/L) | 0.40 ± 0.14 | 0.38 ± 0.12 | 0.39 ± 0.15 | 0.40 ± 0.12 | 0.41 ± 0.13 | 0.44 ± 0.15 |

Data are number (percentage) or mean ± standard deviation.

Abbreviations: NLR, neutrophil-lymphocyte ratio, BMI, body mass index; WBC, white blood cell.

**Table S2. Baseline characteristics of study participants by platelets-lymphocyte ratio (PLR) quintiles.**

| Characteristics | Overall | PLR quintiles | | | | |
| --- | --- | --- | --- | --- | --- | --- |
|  |  | Q1 (<87.11) | Q2 (87.11-104.92) | Q3 (104.92-122.77) | Q4 (122.77-147.40) | Q5 (≥147.40) |
| Number | 96,476 | 19,295 | 19,295 | 19,296 | 19,295 | 19,295 |
| Age (years) | 46.71 ± 13.83 | 49.57 ± 14.87 | 46.81 ± 13.91 | 45.75 ± 13.51 | 45.49 ± 13.17 | 45.92 ± 13.23 |
| Male (%) | 58,496 (60.62) | 14,570 (75.51) | 13,015 (67.45) | 11,818 (61.25) | 10,555 (54.70) | 8,528 (44.20) |
| BMI (kg/m^2^) | 24.22 ± 3.52 | 24.91 ± 3.69 | 24.53 ± 4.03 | 24.24 ± 3.28 | 23.91 ± 3.22 | 23.49 ± 3.11 |
| Ever-smokers (%) | 22,453 (23.27) | 6,179 (32.02) | 5,081 (26.33) | 4,527 (23.46) | 3,774 (19.56) | 2,892 (14.99) |
| Diagnosed hypertension (%) | 29,269 (30.34) | 7,348 (38.08) | 6,147 (31.86) | 5,581 (28.92) | 5,242 (27.17) | 4,951 (25.66) |
| Diagnosed diabetes (%) | 7,346 (7.61) | 2,215 (11.48) | 1,587 (8.22) | 1,352 (7.00) | 1,146 (5.94) | 1,047 (5.43) |
| Diagnosed lung disease (%) | 525 (0.54) | 142 (0.74) | 108 (0.56) | 88 (0.46) | 98 (0.51) | 89 (0.46) |
| Platelets (×10^9^/L) | 223.80 ± 55.05 | 182.91 ± 43.74 | 208.68 ± 42.36 | 223.88 ± 44.48 | 238.53 ± 46.76 | 264.99 ± 58.89 |
| WBC count (×10^9^/L) | 6.07 ± 1.71 | 6.74 ± 2.40 | 6.26 ± 1.43 | 6.03 ± 1.40 | 5.81 ± 1.37 | 5.51 ± 1.43 |
| Neutrophil count (×10^9^/L) | 3.50 ± 1.20 | 3.56 ± 1.37 | 3.50 ± 1.12 | 3.48 ± 1.12 | 3.47 ± 1.13 | 3.48 ± 1.23 |
| Lymphocyte count (×10^9^/L) | 2.00 ± 0.79 | 2.55 ± 1.37 | 2.17 ± 0.44 | 1.97 ± 0.39 | 1.78 ± 0.35 | 1.50 ± 0.34 |
| Monocyte count (×10^9^/L) | 0.40 ± 0.14 | 0.44 ± 0.17 | 0.41 ± 0.13 | 0.40 ± 0.13 | 0.39 ± 0.12 | 0.37 ± 0.12 |

Data are number (percentage) or mean ± standard deviation.

Abbreviations: PLR, platelets-lymphocyte ratio, BMI, body mass index; WBC, white blood cell.

**Table S3. Baseline characteristics of study participants by systemic immune-inflammation index (SII) quintiles.**

| Characteristics | Overall | SII quintiles | | | | |
| --- | --- | --- | --- | --- | --- | --- |
|  |  | Q1 (<262.06) | Q2 (262.06-336.92) | Q3 (336.92-418.21) | Q4 (418.21-540.65) | Q5 (≥540.65) |
| Number | 96,476 | 19,295 | 19,295 | 19,296 | 19,295 | 19,295 |
| Age (years) | 46.71 ± 13.83 | 48.36 ± 14.28 | 46.75 ± 13.75 | 46.02 ± 13.47 | 45.96 ± 13.64 | 46.45 ± 13.87 |
| Male (%) | 58,486 (60.62) | 12,628 (65.45) | 12,336 (63.93) | 12,115 (62.79) | 11,250 (58.31) | 10,157 (52.64) |
| BMI (kg/m^2^) | 24.22 ± 3.52 | 24.02 ± 3.24 | 24.24 ± 3.99 | 24.27 ± 3.27 | 24.31 ± 3.65 | 24.23 ± 3.38 |
| Ever-smokers (%) | 22,453 (23.27) | 4,361 (22.60) | 4,469 (23.16) | 4,693 (24.32) | 4,586 (23.77) | 4,344 (22.51) |
| Diagnosed hypertension (%) | 29,269 (30.34) | 5,714 (29.61) | 5,715 (29.62) | 5,725 (29.67) | 5,929 (30.73) | 6,186 (32.06) |
| Diagnosed diabetes (%) | 7,346 (7.61) | 1,463 (7.58) | 1,391 (7.21) | 1,391 (7.21) | 1,434 (7.43) | 1,667 (8.64) |
| Diagnosed lung disease (%) | 525 (0.54) | 120 (0.62) | 106 (0.55) | 103 (0.53) | 90 (0.47) | 106 (0.55) |
| Platelets (×10^9^/L) | 223.80 ± 55.05 | 182.02 ± 42.44 | 207.23 ± 41.01 | 223.72 ± 43.52 | 239.53 ± 46.27 | 266.50 ± 59.55 |
| WBC count (×10^9^/L) | 6.07 ± 1.71 | 5.35 ± 2.21 | 5.69 ± 1.25 | 5.98 ± 1.31 | 6.29 ± 1.36 | 7.03 ± 1.71 |
| Neutrophil count (×10^9^/L) | 3.50 ± 1.20 | 2.57 ± 1.02 | 3.05 ± 0.71 | 3.40 ± 0.78 | 3.80 ± 0.86 | 4.68 ± 1.31 |
| Lymphocyte count (×10^9^/L) | 2.00 ± 0.79 | 2.24 ± 1.38 | 2.09 ± 0.55 | 2.00 ± 0.53 | 1.91 ± 0.50 | 1.74 ± 0.49 |
| Monocyte count (×10^9^/L) | 0.40 ± 0.14 | 0.37 ± 0.15 | 0.39 ± 0.12 | 0.40 ± 0.12 | 0.41 ± 0.13 | 0.45 ± 0.15 |

Data are number (percentage) or mean ± standard deviation.

Abbreviations: SII, systemic immune-inflammation index, BMI, body mass index; WBC, white blood cell.

**Table S4. Risk of positive nodules by neutrophil-lymphocyte ratio (NLR) quintiles in clinically relevant subgroups.**

| Subgroup | Total (N) | Positive nodules (N) | Q1 (<1.28) | Q2 (1.28-1.57) | Q3 (1.57-1.88) | Q4 (1.88-2.33) | Q5 (≥2.33) | *P* for trend | *P* for interaction |
| --- | --- | --- | --- | --- | --- | --- | --- | --- | --- |
| Age |  |  |  |  |  |  |  |  |  |
| <50 | 60,203 | 4,581 | Ref | 1.11 (1.01-1.22) | 1.11 (1.01-1.22) | 1.10 (0.99-1.21) | 1.16 (1.06-1.28) | 0.0086 | 0.0062 |
| ≥50 | 36,273 | 4,683 | Ref | 0.96 (0.87-1.07) | 1.04 (0.94-1.15) | 1.08 (0.98-1.20) | 1.30 (1.18-1.43) | <0.001 |  |
| Gender |  |  |  |  |  |  |  |  |  |
| Female | 37,990 | 3,616 | Ref | 1.13 (1.01-1.26) | 1.16 (1.04-1.29) | 1.11 (0.99-1.24) | 1.23 (1.11-1.38) | 0.0011 | 0.2154 |
| Male | 58,486 | 5,648 | Ref | 0.97 (0.89-1.07) | 1.01 (0.92-1.10) | 1.04 (0.96-1.14) | 1.15 (1.05-1.25) | 0.0006 |  |
| Smoking status |  |  |  |  |  |  |  |  |  |
| Never | 74,023 | 6,962 | Ref | 1.06 (0.98-1.15) | 1.09 (1.01-1.18) | 1.10 (1.01-1.19) | 1.18 (1.10-1.28) | <0.001 | 0.3951 |
| Ever | 22,453 | 2,302 | Ref | 0.96 (0.83-1.11) | 0.99 (0.86-1.14) | 1.01 (0.88-1.17) | 1.19 (1.04-1.37) | 0.0121 |  |
| BMI |  |  |  |  |  |  |  |  |  |
| <23 | 35,557 | 3,241 | Ref | 0.98 (0.87-1.10) | 1.05 (0.93-1.17) | 1.05 (0.94-1.18) | 1.07 (0.95-1.19) | 0.1181 | 0.0559 |
| ≥23 | 60,919 | 6,023 | Ref | 1.07 (0.98-1.17) | 1.08 (0.99-1.18) | 1.10 (1.00-1.20) | 1.27 (1.17-1.38) | <0.001 |  |

Logistic regression models were used to estimate odds ratios (ORs) and 95% confidence intervals (CIs). Multivariable model was adjusted for age, gender, smoking status, BMI, diagnosed hypertension, diagnosed diabetes and diagnosed lung diseases.

**Table S5. Risk of positive nodules by platelets-lymphocyte ratio (PLR) quintiles in clinically relevant subgroups.**

| Subgroup | Total (N) | Positive nodules (N) | Q1 (<87.11) | Q2 (87.11-104.92) | Q3 (104.92-122.77) | Q4 (122.77-147.40) | Q5 (≥147.40) | *P* for trend | *P* for interaction |
| --- | --- | --- | --- | --- | --- | --- | --- | --- | --- |
| Age |  |  |  |  |  |  |  |  |  |
| <50 | 60,203 | 4,581 | Ref | 1.05 (0.95-1.16) | 0.99 (0.90-1.10) | 1.09 (0.99-1.20) | 1.06 (0.96-1.17) | 0.2099 | 0.4202 |
| ≥50 | 36,273 | 4,683 | Ref | 1.00 (0.91-1.09) | 1.08 (0.98-1.18) | 1.12 (1.02-1.23) | 1.15 (1.05-1.27) | 0.0003 |  |
| Gender |  |  |  |  |  |  |  |  |  |
| Female | 37,990 | 3,616 | Ref | 1.03 (0.90-1.17) | 1.10 (0.97-1.25) | 1.16 (1.03-1.31) | 1.10 (0.98-1.24) | 0.0189 | 0.5570 |
| Male | 58,486 | 5,648 | Ref | 1.05 (0.97-1.14) | 1.04 (0.95-1.13) | 1.11 (1.02-1.21) | 1.13 (1.03-1.23) | 0.0045 |  |
| Smoking status | |  |  |  |  |  |  |  |  |
| Never | 74,023 | 6,962 | Ref | 1.07 (0.99-1.17) | 1.09 (1.00-1.18) | 1.17 (1.08-1.27) | 1.15 (1.06-1.24) | 0.0001 | 0.4348 |
| Ever | 22,453 | 2,302 | Ref | 0.99 (0.87-1.12) | 1.00 (0.88-1.14) | 1.03 (0.90-1.18) | 1.02 (0.89-1.19) | 0.5638 |  |
| BMI |  |  |  |  |  |  |  |  |  |
| <23 | 35,557 | 3,241 | Ref | 1.07 (0.95-1.22) | 1.00 (0.88-1.14) | 1.14 (1.01-1.29) | 1.08 (0.96-1.22) | 0.1146 | 0.4635 |
| ≥23 | 60,919 | 6,023 | Ref | 1.03 (0.95-1.12) | 1.09 (1.01-1.19) | 1.13 (1.04-1.23) | 1.15 (1.05-1.25) | 0.0003 |  |

Logistic regression models were used to estimate odds ratios (ORs) and 95% confidence intervals (CIs). Multivariable model was adjusted for age, gender, smoking status, BMI, diagnosed hypertension, diagnosed diabetes and diagnosed lung diseases.

**Table S6. Risk of positive nodules by systemic immune-inflammation index (SII) quintiles in clinically relevant subgroups.**

| Subgroup | Total (N) | Positive nodules (N) | Q1 (<262.06) | Q2 (262.02-336.92) | Q3 (336.92-418.21) | Q4 (418.21-540.65) | Q5 (≥540.65) | *P* for trend | *P* for interaction |
| --- | --- | --- | --- | --- | --- | --- | --- | --- | --- |
| Age |  |  |  |  |  |  |  |  |  |
| <50 | 60,203 | 4,581 | Ref | 0.96 (0.87-1.07) | 1.09 (0.99-1.21) | 1.04 (0.94-1.15) | 1.05 (0.95-1.16) | 0.1048 | 0.0112 |
| ≥50 | 36,273 | 4,683 | Ref | 0.99 (0.90-1.09) | 0.95 (0.87-1.05) | 1.11 (1.01-1.22) | 1.17 (1.06-1.28) | <0.001 |  |
| Gender |  |  |  |  |  |  |  |  |  |
| Female | 37,990 | 3,616 | Ref | 0.98 (0.87-1.10) | 1.14 (1.02-1.27) | 1.11 (0.99-1.24) | 1.08 (0.97-1.21) | 0.0267 | 0.0899 |
| Male | 58,486 | 5,648 | Ref | 0.99 (0.91-1.08) | 0.99 (0.90-1.07) | 1.07 (0.98-1.17) | 1.12 (1.02-1.22) | 0.0027 |  |
| Smoking status | |  |  |  |  |  |  |  |  |
| Never | 74,023 | 6,962 | Ref | 1.00 (0.93-1.08) | 1.06 (0.98-1.15) | 1.09 (1.01-1.18) | 1.10 (1.01-1.18) | 0.0029 | 0.6896 |
| Ever | 22,453 | 2,302 | Ref | 0.95 (0.82-1.09) | 0.99 (0.87-1.14) | 1.08 (0.94-1.24) | 1.13 (0.98-1.29) | 0.0165 |  |
| BMI |  |  |  |  |  |  |  |  |  |
| <23 | 35,557 | 3,241 | Ref | 0.93 (0.83-1.04) | 1.05 (0.94-1.18) | 1.03 (0.92-1.15) | 1.04 (0.93-1.16) | 0.1657 | 0.3528 |
| ≥23 | 60,919 | 6,023 | Ref | 1.02 (0.94-1.12) | 1.04 (0.96-1.14) | 1.13 (1.04-1.23) | 1.15 (1.06-1.25) | 0.0001 |  |

Logistic regression models were used to estimate odds ratios (ORs) and 95% confidence intervals (CIs). Multivariable model was adjusted for age, gender, smoking status, BMI, diagnosed hypertension, diagnosed diabetes and diagnosed lung diseases.

**Table S7. Baseline characteristics of participants with lung cancer detected by CT screening.**

|  | Total (N = 96,476) | Without lung cancer (N = 95,907) | With lung cancer (n=569) | *P* value |
| --- | --- | --- | --- | --- |
| Age (years) | 46.71 ± 13.83 | 46.66 ± 13.81 | 55.68 ± 13.84 | <0.001 |
| <50 | 60,203 (62.40) | 60,007 (62.40) | 196 (34.45) | <0.001 |
| ≥50 | 36,273 (37.60) | 35,900 (37.43) | 373 (65.55) |  |
| Gender |  |  |  |  |
| Female | 37,990 (39.38) | 37,710 (39.32) | 280 (49.21) | <0.001 |
| Male | 58,486 (60.62) | 58,197 (60.68) | 289 (50.79) |  |
| BMI (Kg/m^2^) |  |  |  |  |
| ≤18.4 | 2,324 (2.41) | 2,305 (2.40) | 19 (3.34) | 0.085 |
| 18.5-22.9 | 33,233 (34.45) | 33,020 (34.43) | 213 (37.43) |  |
| ≥23.0 | 60,919 (63.14) | 60,582 (63.17) | 337 (59.23) |  |
| Smoking status |  |  |  |  |
| Never | 74,023 (76.73) | 73,579 (76.72) | 444 (78.03) | 0.460 |
| Ever | 22,453 (23.27) | 22,328 (23.28) | 125 (21.97) |  |
| SBP (mm Hg) | 126.59 ± 17.81 | 126.57 ± 17.79 | 130.50 ± 19.92 | <0.001 |
| DBP (mm Hg) | 77.44 ± 11.31 | 77.44 ± 11.31 | 76.96 ± 11.50 | 0.316 |
| Diagnosed hypertension |  |  |  |  |
| No | 67,207 (69.66) | 66,872 (69.73) | 335 (58.88) | <0.001 |
| Yes | 29,269 (30.34) | 29,035 (30.27) | 234 (41.12) |  |
| Diagnosed diabetes |  |  |  |  |
| No | 89,130 (92.39) | 88,622 (92.40) | 508 (89.28) | 0.005 |
| Yes | 7,346 (7.61) | 7,285 (7.60) | 61 (10.72) |  |
| Diagnosed lung disease |  |  |  |  |
| No | 95,951 (99.46) | 95,386 (99.46) | 565 (99.30) | 0.606 |
| Yes | 525 (0.54) | 521 (0.54) | 4 (0.70) |  |
| Platelets (×10^9^/L) | 223.80 ± 55.05 | 223.82 ± 55.03 | 220.72 ± 58.37 | 0.181 |
| WBC count (×10^9^/L) | 6.07 ± 1.71 | 6.07 ± 1.71 | 6.00 ± 1.50 | 0.351 |
| Neutrophil count (×10^9^/L) | 3.50 ± 1.20 | 3.50 ± 1.20 | 3.53 ± 1.18 | 0.496 |
| Lymphocyte count (×10^9^/L) | 2.00 ± 0.79 | 2.00 ± 0.79 | 1.92 ± 0.59 | 0.022 |
| Monocyte count (×10^9^/L) | 0.40 ± 0.14 | 0.40 ± 0.14 | 0.39 ± 0.14 | 0.010 |
| NLR | 1.87 ± 0.79 | 1.86 ± 0.79 | 1.99 ± 0.91 | <0.001 |
| PLR | 119.43 ± 40.25 | 119.41 ± 40.18 | 123.73 ± 49.91 | 0.011 |
| SII | 417.22 ± 211.32 | 417.06 ± 210.48 | 443.28 ± 321.47 | 0.003 |

Data are number (percentage) or mean ± standard deviation.

Abbreviations: BMI, body mass index; SBP, Systolic blood pressure; DBP, Diastolic blood pressure; WBC, White blood cell; NLR, neutrophil-to-lymphocyte ratio; PLR, platelet to lymphocyte ratio; SII, systemic immune-inflammation index.

**Table S8. Risk of lung cancer by neutrophil-lymphocyte ratio (NLR) quintiles in clinically relevant subgroups.**

| Subgroup | Total (N) | Lung cancer (N) | Q1 (<1.28) | Q2 (1.28-1.57) | Q3 (1.57-1.88) | Q4 (1.88-2.33) | Q5 (≥2.33) | *P* for quadratic term | *P* for interaction |
| --- | --- | --- | --- | --- | --- | --- | --- | --- | --- |
| Age |  |  |  |  |  |  |  |  |  |
| <50 | 60,203 | 196 | 0.99 (0.62-1.59) | 1.38 (0.89-2.13) | Ref | 0.88 (0.55-1.42) | 1.34 (0.86-2.07) | 0.5543 | 0.2575 |
| ≥50 | 36,273 | 373 | 1.22 (0.87-1.72) | 0.97 (0.67-1.39) | Ref | 1.04 (0.73-1.48) | 1.55 (1.13-2.12) | 0.0034 |  |
| Gender |  |  |  |  |  |  |  |  |  |
| Female | 37,990 | 280 | 1.10 (0.77-1.58) | 0.87 (0.59-1.28) | Ref | 0.72 (0.48-1.08) | 1.16 (0.82-1.66) | 0.0491 | 0.1403 |
| Male | 58,486 | 289 | 1.23 (0.81-1.87) | 1.48 (0.99-2.21) | Ref | 1.30 (0.87-1.95) | 1.66 (1.14-2.42) | 0.1378 |  |
| Smoking status |  |  |  |  |  |  |  |  |  |
| Never | 74,023 | 444 | 1.21 (0.90-1.65) | 1.08 (0.78-1.48) | Ref | 1.03 (0.75-1.41) | 1.39 (1.04-1.87) | 0.0168 | 0.5690 |
| Ever | 22,453 | 125 | 0.94 (0.50-1.77) | 1.32 (0.75-2.33) | Ref | 0.79 (0.42-1.46) | 1.33 (0.78-2.52) | 0.6136 |  |
| BMI |  |  |  |  |  |  |  |  |  |
| <23 | 35,557 | 232 | 1.21 (0.80-1.84) | 1.07 (0.70-1.84) | Ref | 0.94 (0.60-1.47) | 1.29 (0.86-1.94) | 0.1070 | 0.8628 |
| ≥23 | 60,919 | 337 | 1.11 (0.77-1.60) | 1.16 (0.81-1.65) | Ref | 1.00 (0.70-1.44) | 1.50 (1.08-2.08) | 0.0607 |  |

Logistic regression models were used to estimate odds ratios (ORs) and 95% confidence intervals (CIs). Multivariable model was adjusted for age, gender, smoking status, BMI, diagnosed hypertension, diagnosed diabetes and diagnosed lung diseases.

**Table S9. Risk of lung cancer by platelets-lymphocyte ratio (PLR) quintiles in clinically relevant subgroups.**

| Subgroup | Total (N) | Lung cancer (N) | Q1 (<87.11) | Q2 (87.11-104.92) | Q3 (104.92-122.77) | Q4 (122.77-147.40) | Q5 (≥147.40) | *P* for trend | *P* for interaction |
| --- | --- | --- | --- | --- | --- | --- | --- | --- | --- |
| Age |  |  |  |  |  |  |  |  |  |
| <50 | 60,203 | 196 | Ref | 1.44 (0.84-2.46) | 1.62 (0.97-2.72) | 1.17 (0.68-2.01) | 1.41 (0.84-2.38) | 0.4065 | 0.2935 |
| ≥50 | 36,273 | 373 | Ref | 1.03 (0.75-1.41) | 0.96 (0.69-1.33) | 1.14 (0.83-1.57) | 1.23 (0.90-1.68) | 0.1508 |  |
| Gender |  |  |  |  |  |  |  |  |  |
| Female | 37,990 | 280 | Ref | 1.10 (0.72-1.69) | 0.89 (0.58-1.38) | 1.17 (0.78-1.75) | 1.15 (0.78-1.70) | 0.4482 | 0.3026 |
| Male | 58,486 | 289 | Ref | 1.20 (0.85-1.70) | 1.47 (1.04-2.08) | 1.09 (0.75-1.61) | 1.37 (0.95-1.98) | 0.2112 |  |
| Smoking status |  |  |  |  |  |  |  |  |  |
| Never | 74,023 | 444 | Ref | 1.21 (0.88-1.65) | 1.12 (0.82-1.54) | 1.15 (0.84-1.58) | 1.22 (0.90-1.65) | 0.3152 | 0.7189 |
| Ever | 22,453 | 125 | Ref | 1.03 (0.60-1.77) | 1.38 (0.82-2.31) | 1.18 (0.86-2.07) | 1.49 (0.86-2.58) | 0.1451 |  |
| BMI |  |  |  |  |  |  |  |  |  |
| <23 | 35,557 | 232 | Ref | 0.96 (0.61-1.52) | 1.09 (0.70-1.70) | 1.08 (0.70-1.67) | 1.13 (0.75-1.72) | 0.4400 | 0.8694 |
| ≥23 | 60,919 | 337 | Ref | 1.30 (0.93-1.81) | 1.25 (0.89-1.76) | 1.24 (0.87-1.76) | 1.44 (1.02-2.03) | 0.0854 |  |

Logistic regression models were used to estimate odds ratios (ORs) and 95% confidence intervals (CIs). Multivariable model was adjusted for age, gender, smoking status, BMI, diagnosed hypertension, diagnosed diabetes and diagnosed lung diseases.

**Table S10. Risk of lung cancer by systemic immune-inflammation index (SII) quintiles in clinically relevant subgroups.**

| Subgroup | Total (N) | Lung cancer (N) | Q1 (<262.06) | Q2 (262.02-336.92) | Q3 (336.92-418.21) | Q4 (418.21-540.65) | Q5 (≥540.65) | *P* for trend | *P* for interaction |
| --- | --- | --- | --- | --- | --- | --- | --- | --- | --- |
| Age |  |  |  |  |  |  |  |  |  |
| <50 | 60,203 | 196 | Ref | 0.76 (0.46-1.26) | 1.27 (0.81-1.98) | 0.83 (0.51-1.34) | 1.10 (0.70-1.73) | 0.5887 | 0.2210 |
| ≥50 | 36,273 | 373 | Ref | 1.23 (0.89-1.69) | 1.11 (0.80-1.55) | 1.19 (0.85-1.65) | 1.48 (1.08-2.02) | 0.0385 |  |
| Gender |  |  |  |  |  |  |  |  |  |
| Female | 37,990 | 280 | Ref | 0.92 (0.62-1.38) | 1.24 (0.85-1.81) | 1.11 (0.76-1.62) | 1.17 (0.81-1.69) | 0.2422 | 0.4907 |
| Male | 58,486 | 289 | Ref | 1.22 (0.85-1.75) | 1.19 (0.82-1.72) | 1.00 (0.68-1.48) | 1.49 (1.04-2.12) | 0.1474 |  |
| Smoking status |  |  |  |  |  |  |  |  |  |
| Never | 74,023 | 444 | Ref | 1.06 (0.78-1.45) | 1.30 (0.96-1.75) | 1.11 (0.82-1.51) | 1.38 (1.03-1.85) | 0.0406 | 0.7454 |
| Ever | 22,453 | 125 | Ref | 1.13 (0.65-1.96) | 0.96 (0.54-1.70) | 0.91 (0.51-1.62) | 1.14 (0.67-1.96) | 0.9286 |  |
| BMI |  |  |  |  |  |  |  |  |  |
| <23 | 35,557 | 232 | Ref | 0.99 (0.64-1.51) | 1.27 (0.85-1.90) | 1.04 (0.68-1.58) | 1.32 (0.89-1.96) | 0.1917 | 0.9562 |
| ≥23 | 60,919 | 337 | Ref | 1.13 (0.80-1.61) | 1.18 (0.83-1.68) | 1.09 (0.77-1.56) | 1.37 (0.98-1.91) | 0.1285 |  |

Logistic regression models were used to estimate odds ratios (ORs) and 95% confidence intervals (CIs). Multivariable model was adjusted for age, gender, smoking status, BMI, diagnosed hypertension, diagnosed diabetes and diagnosed lung diseases.
