# Supplementary figures and images for: Associations of systemic inflammation markers with identification of pulmonary nodule and incident lung cancer in Chinese population
Source: Cancer Med. 2022 Apr 5;11(12):2482–91. doi: 10.1002/cam4.4606 (PMC9189452; doi:10.1002/cam4.4606)

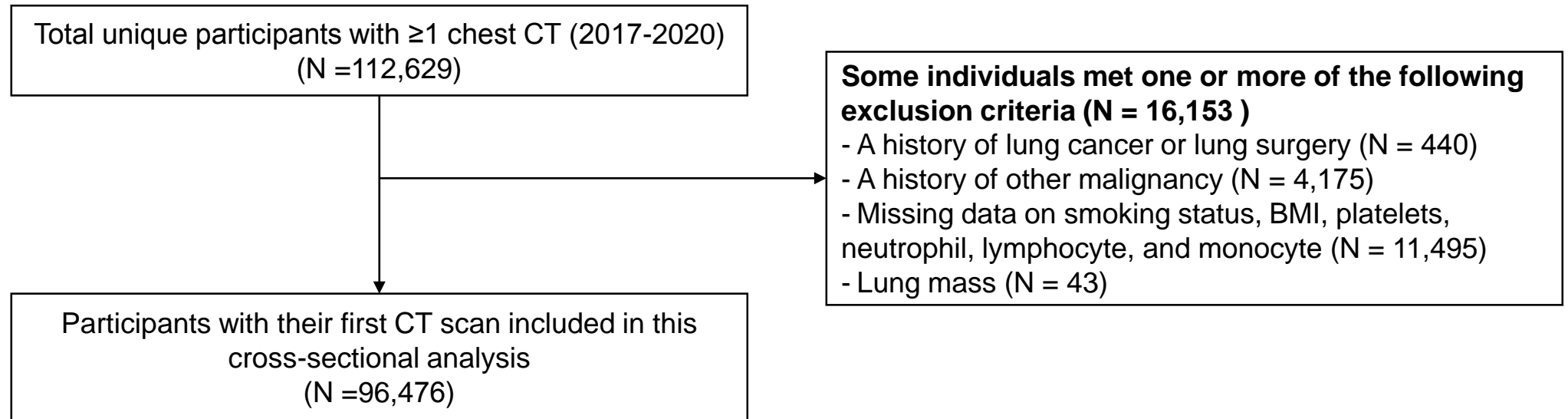

Supplement: Supplementary file 2 — Figure S1 [file CAM4-11-2482-s003.pdf]

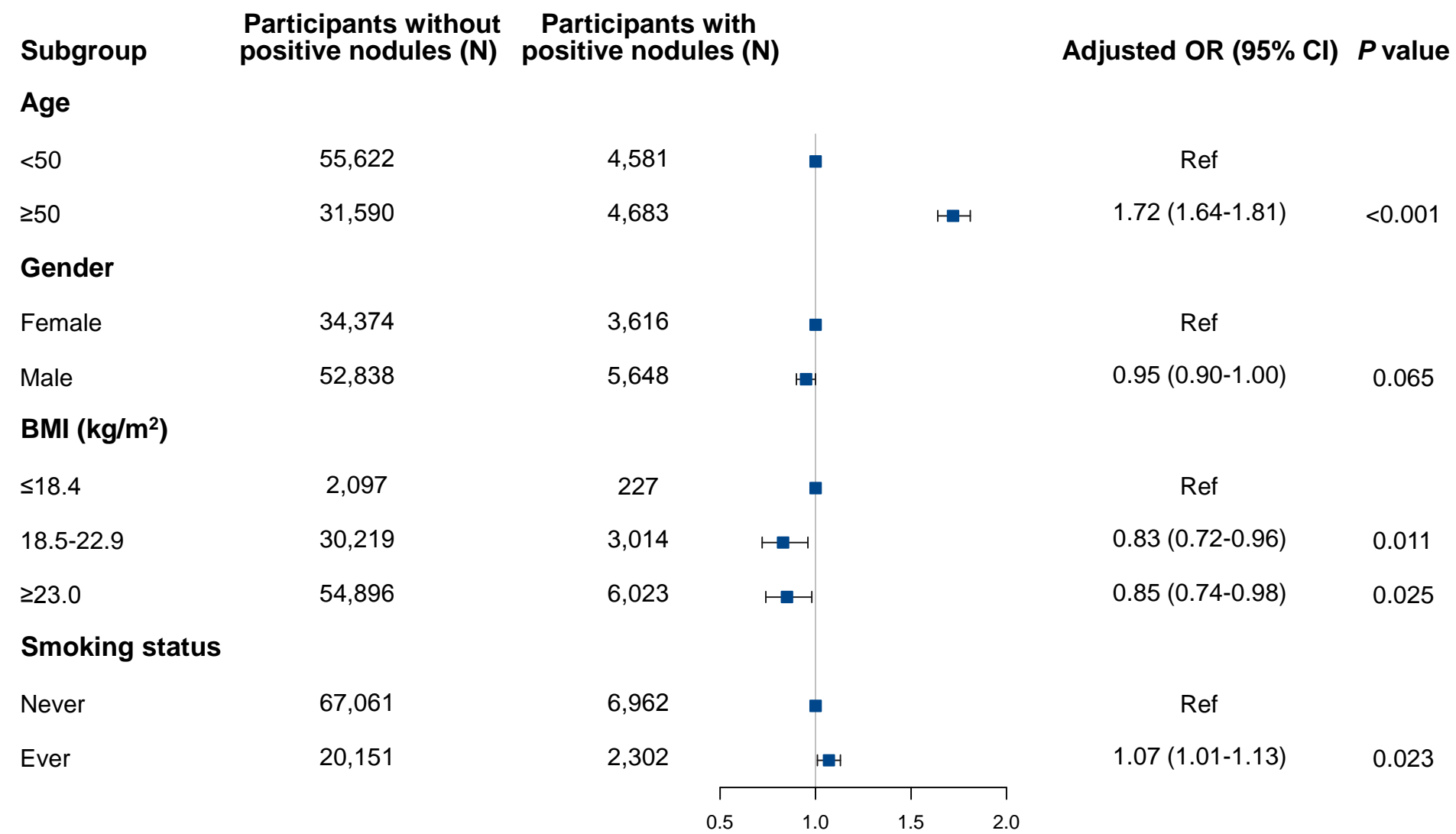

Supplement: Supplementary file 3 — Figure S2 [file CAM4-11-2482-s002.pdf]
